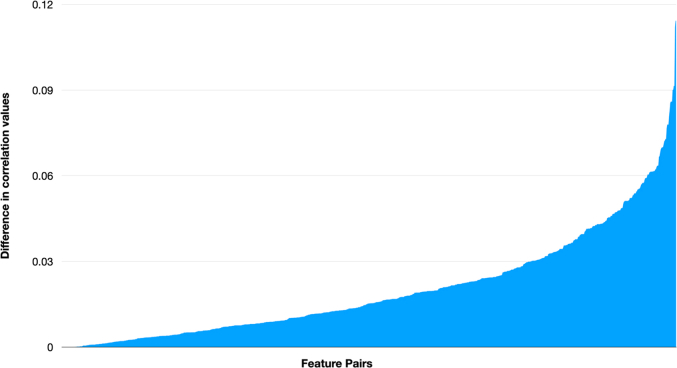# NFnetFu: A novel workflow for microbiome data fusion

**DOI:** 10.1016/j.compbiomed.2021.104556

**Published:** 2021-08

**Authors:** Vartika Bisht, Animesh Acharjee, Georgios V. Gkoutos

**Affiliations:** a College of Medical and Dental Sciences, Institute of Cancer and Genomic Sciences, Centre for Computational Biology, University of Birmingham, B15 2TT, UK; b Institute of Translational Medicine, University Hospitals Birmingham NHS, Foundation Trust, B15 2TT, UK; c NIHR Surgical Reconstruction and Microbiology Research Centre, University Hospital Birmingham, Birmingham, B15 2WB, UK; d MRC Health Data Research UK HDR, UK; e NIHR Experimental Cancer Medicine Centre, B15 2TT, Birmingham, UK; f NIHR Biomedical Research Centre, University Hospital Birmingham, Birmingham, B15 2TT, UK

**Keywords:** Microbiome, Fuzzy inference, Clustering, Network fusion

## Abstract

Microbiome data analysis and its interpretation into meaningful biological insights remain very challenging for numerous reasons, perhaps most prominently, due to the need to account for multiple factors, including collinearity, sparsity (excessive zeros) and effect size, that the complex experimental workflow and subsequent downstream data analysis require. Moreover, a meaningful microbiome data analysis necessitates the development of interpretable models that incorporate inferences across available data as well as background biomedical knowledge. We developed a multimodal framework that considers sparsity (excessive zeros), lower effect size, intrinsically microbial correlations, i.e., collinearity, as well as background biomedical knowledge in the form of a cluster-infused enriched network architecture. Finally, our framework also provides a candidate taxa/Operational Taxonomic Unit (OTU) that can be targeted for future validation experiments. We have developed a tool, the term NFnetFU (Neuro Fuzzy network Fusion), that encompasses our framework and have made it freely available at https://github.com/VartikaBisht6197/NFnetFu.

## Highlights

• Microbiome data set have many challenges including collinearity, sparsity (excessive zeros) and effect size.
• A sequential workflow and downstream data analysis is required to capture knowledge from each step.
• We developed a microbiome enrichment based fusion score called “*NFnetFu”* to facilitate data analysis.

## Graphical abstract

**Figure:**
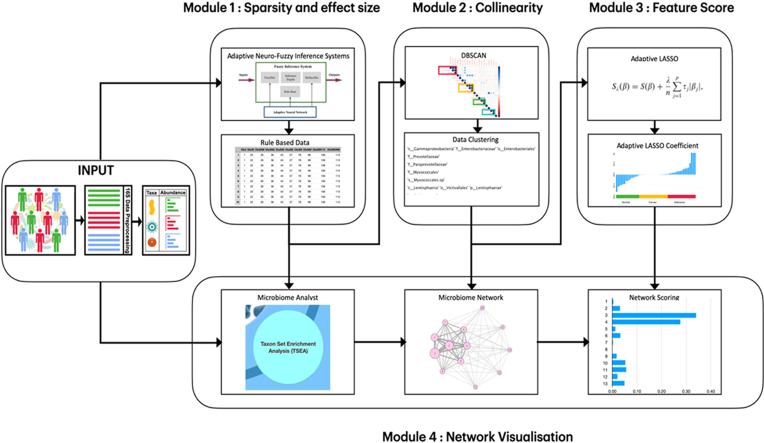

## 1 Introduction

A myriad of microorganisms, bacteria, viruses, and fungi, are abandoned within an organism, comprising its so-called microbiome. Different organ systems are characterized by distinct microbiota populations, which are now widely recognized as contributors to phenotypic manifestations across all organisms. Most notably, the microbiota host interactions of the gastrointestinal tract in humans are now understood to influence large aspects of the human biology repertoire [1].

Microbial imbalance, caused by a variety of factors, most prominent environmental ones, can have a significant effect on an organism's pathobiology and pathophysiology [[2], [3]]. It is now understood that microbiota-host interactions affect the manifestation, development, and progression of major diseases, including autoimmune disorders [4] respiratory diseases [5,6], inflammation disease [6,7] cancer [[8], [9], [10], [11]], metabolic diseases [[12], [13], [14], [15], [16]], liver diseases [17,18], as well as behaviour related disease and disorders [19]. The microbiome of an organism influences its physiology, regulates several of its complex biological processes and affects several important host functions, such as digestion, enzyme and vitamin production, as well as host immune system modulation via complex metabolic interactions [20,21]. Many studies reported the complex interplay of the microbiome within the ‘omes puzzle [22,23] and the large-scale dynamics that govern it. In an effort to decipher them, several computational approaches and workflows have been proposed and developed in recent years [24,25].

These approaches can be broadly categorized in two types. The first one focuses on the processing and quality control of microbiome data resulting from either 16s rRNA gene sequencing [26,27] or metagenomics experiments [28,29]. Examples of such approaches include MetAMOS, a fully automated metagenomic analysis platform, which covers the whole spectrum that ranges from next-generation sequencing reads to functional annotations [30]. MetAMOS provides an automated platform for the analysis of metagenomic datasets providing systematic gene predictions outputs. Comeau et al., 2017 [31] developed a step-by-step custom gene sequencing protocol emphasizing on the fast and reliable microbiome analysis that will allow microbiome researchers to focus more on potential future experiment designs. Other modular-based approaches, such as MicrobiomeAnalyst [32,33], form standalone microbiome analysis web tools that contain modules for marker-gene data profiling, shotgun data profiling, and Taxon Set Enrichment Analysis (TSEA).

The second type of approach revolves around the workflows typically designed for downstream analysis of taxonomic data. One such example is tmap, a network-based stratification tool using high-dimensional microbiome data [34]. tmap employs network-based topological data analysis and caters the stratification of microbiome population, as well as microbiome data associations, based network-based representations. This method utilizes advanced large-scale data mining techniques to identify the association of taxa (Operational Taxonomic Unit, OTU. Other examples, such as the Linear Discriminant Analysis (LDA) effect size (LEfSe) method, concentrate on the metagenomic biomarker discovery [35]. LEfSe determines the variables/taxa most likely to explain differences between classes (outcome variables) by using statistical tests and biological relevance. Finally, other methods, for example MetaBoot [36] exploit bootstrapping frequency to discover taxonomical biomarkers for different microbial communities based on metagenomic data.

Despite these recent developments though, microbiome data analysis and its interpretation into meaningful biological insights remains very challenging for several reasons, most prominently due to the complex experimental workflows and subsequent downstream data analysis the interpretation of such data necessitates. Some of the challenges that need to be addressed include the fact that microbiome data are highly sparse i.e., they contain many zeros across samples and taxa [37,38]. Thorsen et al. [39] developed a large-scale benchmarking tool which revealed that typically relative differential abundance tools are sensitive to sparsity. Another challenge relates to the microbial data multicollinearity generating complex covariance structures. Such multicollinearity leads to several challenges in model building as well as in the estimation over large numbers of unstable coefficients or weights that overfit the data and do not generalize over new datasets [40]. Moreover, microbes are intrinsically associated or linked as part of their interactions. Furthermore, the effect size of the microbiome's data is relatively small compared to other types of omics datasets, for example gene expression or metabolomics data, that render their modelling complicated [41]. Finally, a meaningful microbiome data analysis necessitates the development of interpretable models that consider both compositional data as well as biomedical knowledge inferences.

So as to address these challenges, we developed a novel downstream microbiome analysis framework that accounts for collinearity, sparsity and effect size. Our framework includes several modules, developed to address different microbiome data analysis challenges. Module 1 focuses on the sparsity and effect size, Module 2 addresses collinearity while Module 3 concentrates on network fusion i.e., the combination of the data and biological driven knowledge deriving an interpretable score. We used three different datasets to assess our workflow and we validated our approach using published literature discussing involvement of selected microbes with diseases.

## 2 Results

**Fig. 1:**
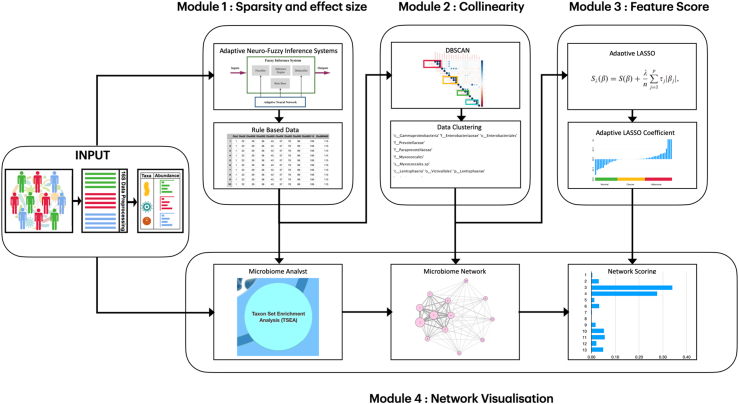
A schematic diagram of the framework modularisation to address sparsity, collinearity, effect size, and finally the fusion of the data and experimentally derived biological networks.

We have developed a framework, NFnetFu, for microbiome data analysis that accounts for sparsity, microbiome features' small effect size and collinearly and then enables microbiome based enrichment analysis. One of NFnetFu's novel features lies with its transformation of microbiome profiles into a network representation that captures and prioritises microbes and their interactions. The framework is divided into different modules, outlined in Fig. 1, and discussed in detail in the methods section.

### 2.1 Module 1: application of adaptive neuro-fuzzy inference system (ANFIS) to overcome sparsity and small effect size

Sparsity is a frequent feature of 16s rRNA and metagenomics datasets. This module applies a supervised adaptive neuro-fuzzy inference system (ANFIS) [42] algorithm to learn the input matrix and outputs a rule-based inference matrix. The resulting matrix is then processed for further downstream analysis. The conversion to a rule-based matrix preserves the intra-feature correlation. Table 1 describes the application of the ANFIS application on different datasets. The learning algorithm not only takes into account different effect sizes but also considers potential feature associations with the output labels (for example PSC-UC, UC, and Healthy Controls, Dataset 1). The rows in the inferred matrix correspond to the inference rules (referred to as “effective samples'') as opposed to the input matrix, which corresponds to the patient samples. The number of entries in the Dataset 2 input matrix is higher than the ones in Dataset 3. Counter intuitively, in Dataset 3, a reduction from the number of samples to the number of effective samples is observed, even though the Dataset 1 samples number is higher than the Dataset 3 one. Since the algorithm relies on a neural network-based approach, the interpretation of the cause of a decision is not possible. The resulting matrix (Module 1) is centered and scaled (i.e., auto-scaled) for further analysis. Supplementary Table 1a depicts the correlation matrix of the abundance matrix and the Supplementary Table 1b presents the correlation matrix of the rule based matrix for Dataset 1. Similar matrices for Datasets 2 and 3 can be found in the Supplementary Tables 2a, 2b and 3a, 3b respectively. Comparing each entry in these matrices, based on the corresponding feature pair, the absolute difference of correlation values at each point was calculated. We then compared the differences between correlation values calculated before and after the application of ANFIS for each feature pair. Supplementary Fig. 1 represents the absolute difference between the features for Dataset 1. The minimum and maximum absolute difference between feature pairs is 0 and 0.11 respectively. Similar plots for Datasets 2 and 3 can be found in the Supplementary Figs. 2 and 3. The maximum absolute difference for Dataset 2 and 3 was 0.098 and 0.167 respectively. This indicates that the correlation structure of the matrix before and after the ANFIS application was conserved.

**Table 1:** ANFIS application on the different datasets. For each dataset, the change in the number of samples before and after ANFIS is different. For Dataset 1 and 2, unlike Dataset 3, there is no difference between the number of samples.

Dataset	No. of samples before ANFIS	No. of sample after ANFIS	Output rule based matrix
1	30	30	Supplementary Table 4a
2	490	490	Supplementary Table 4b
3	422	355	Supplementary Table 4c

### 2.2 Module 2: reducing collinearity using the density-based clustering (DBSCAN) method

This module clusters highly collinear microbiome features in the rule-based matrix (Module 1). A density-based clustering method, DBSCAN [43], is applied to cluster these microbiome features. The DBSCAN algorithm requires two parameters, namely the value of epsilon, to define a neighbourhood (eps), and the minimum number of features in the epsilon neighbourhood (minpt), to cluster the features. The module computes an appropriate epsilon value for the input rule-based matrix and clusters together features in the overlapping epsilon neighbourhoods. Table 2 presents the different numbers of clusters and values of epsilon across the different datasets. Supplementary Tables 1b and 1e shows the correlation matrix for Dataset 1 input rule-based matrix and resultant matrix following the application of Module 2. Supplementary Tables 1c and 1f shows the p values for respective matrices. Groups of significant highly collinear features (p cutoff 0.05) along the diagonal (Supplementary Table 1b) are clustered together, resulting in a matrix with low collinearity (Supplementary Table 1d). Similar tables for the Datasets 2 and 3 can be found in the Supplementary Tables 2b,2c,2e,2f and 3b,3c,3e,3f respectively.

**Table 2:** Groupings (or clustering) differences across the different datasets. A higher number of Dataset 1 highly collinear features groups is observed in relation to the Dataset 2 and 3 ones.

Dataset	No. of groups	Maximum no. of features in a group	Value of epsilon	No. of features in the input dataset	No. of features in the resulting dataset
1	13	4	3.5	47	26
2	1	3	11	100	98
3	1	2	5.5	100	99

### 2.3 Module 3: adaptive LASSO (Least Absolute Shrinkage and Selection Operator) based feature score calculation

**Fig. 2:**
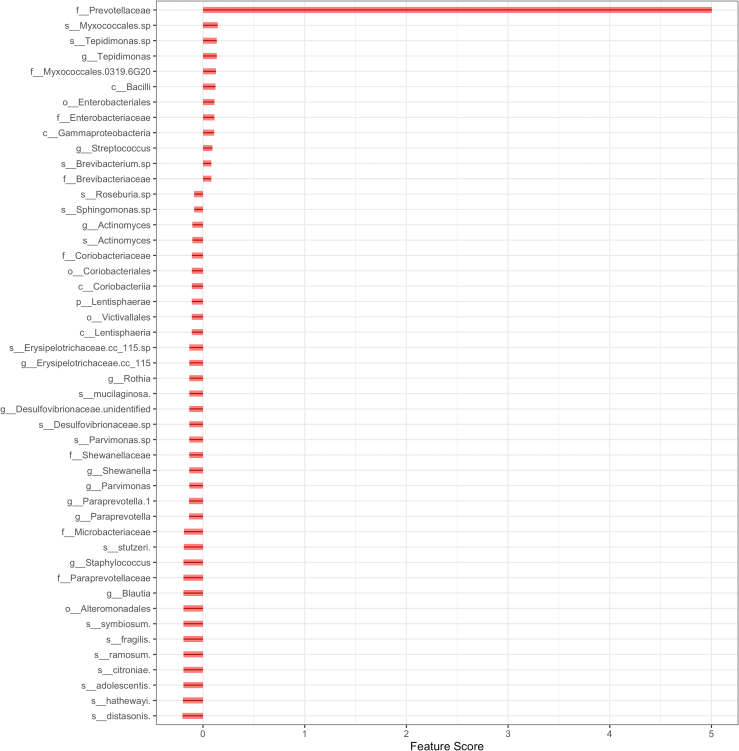
A bar plot depicting the feature scores of the Dataset 1 microbiome features. The feature score for microbe *f__Prevotellaceae* corresponding to *Prevotellaceae* family is 5 while the *s__distasonis* corresponding to species *Distasonis* score is −2. These scores indicate the association of the microbiome features with the outcome variable. The scores are employed to prioritize microbes in the network which can then be applied for potential targeted research.

The microbiome features scores are then calculated, which eventually aid the microbe prioritization. The scores are calculated using adaptive LASSO. Adaptive LASSO (Least Absolute Shrinkage and Selection Operator) [44] is a variation of the original LASSO technique [45] with oracle properties i.e. simultaneous, consistent variable selection [46] and optimal variable estimation. Fig. 2 presents the different microbe feature scores (Dataset 1). Microbe *f__Prevotellaceae*, corresponding to family *Prevotellaceae*, is assigned with the highest feature score. The distribution of feature score is skewed towards *f__Prevotellaceae (Skewness*: 6.601666 and *Kurtosis*: 44.605896). Supplementary Fig. 5 presents a bar plot for the Dataset 3 feature scores. Dataset 3 results reveal that the distribution of feature scores is skewed (*Skewness*: 9.649418 and *Kurtosis*: 95.4272) towards the OTU corresponding to the genus *Lactobacillus* (OTU00001) with the highest feature score. Supplementary Fig. 4 presents a bar plot of Dataset 2 feature scores. The feature scores vary from 3.9 to −3.6 and the distribution is fairly symmetric (*Skewness*: −0.450515 and *Kurtosis*: 5.268463). These scores correspond to the feature and outcome variable association. The skewness allows for a better prioritization of microbes while catering the selection of potential microbes that can be used for designing future microbiome targeted therapeutics studies. Fig. 2. A bar plot depicting the Dataset 1 microbiome feature scores. The feature score for microbe *f__Prevotellaceae* corresponding to the *Prevotellaceae* family is 5 while the *s__distasonis* score corresponding to the species *Distasonis* score is −2. These scores indicate an association of the microbiome features with the outcome variable. These scores are used to prioritize microbes in the network which can then be used for potential targeted research.

### 2.4 Module 4: cluster-infused TSEA based network fusion

**Fig. 3:**
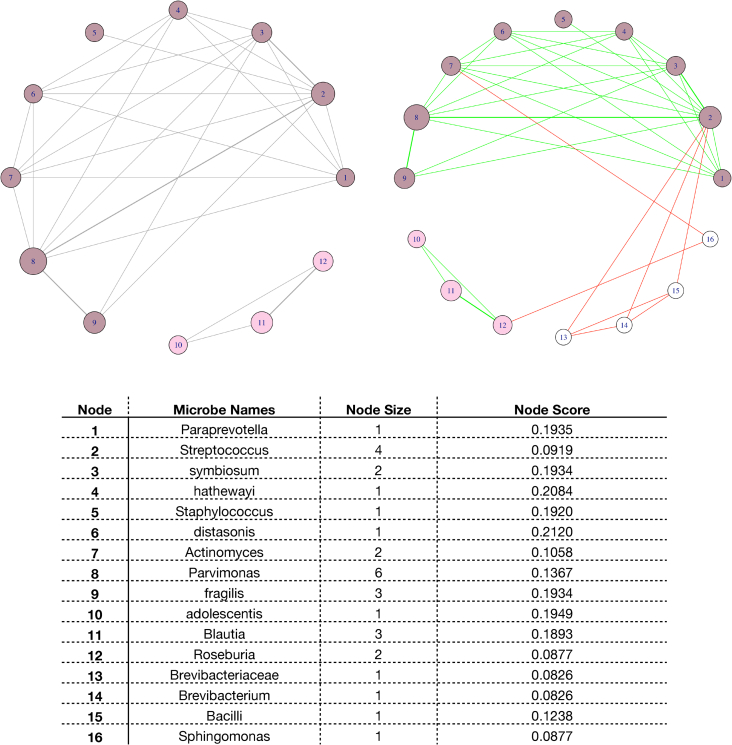
The cluster-infused TSEA based network architecture. a) The node size corresponds to the frequency of occurrence of individual microbes in the TSEA results. The thickness of edges corresponds to the frequency of occurrence of the associated pair of microbes in TSEA results. Nodes 1 to 12 can be found within the TSEA results. This network represents microbial iterations documented in the literature. b). New nodes and edges are added to the biological network, based on the earlier modules' outputs. The network connections are presented in green. The new iterations, resulting from the infusing data-driven results, are depicted in red. The white nodes correspond to the newly added nodes. The node score prioritises microbe *Distasonis* (Dataset 1).

A TSEA-derived biological network is subsequently computed, based on a Taxon Set Enrichment Analysis (TSEA) [32]. The resulting matrix mixed level microbiome feature names (Module 1) are converted into microbe names used by TSEA (Metaboanalyst). Various microbe datasets are integrated based on their converted microbe name. For instance, microbe *Myxococcales* (Dataset 1) is the result of the combination *f__Myxococcales.0319.6G20* and *s__Myxococcales.sp,* belonging to the order *Myxococcales* [47]. These microbiome features were clustered together (Module 2), using an independent method, which caters for a density-based clustering. TSEA takes as input a list of converted microbe names and uses them for enrichment over published literature data. The node size corresponds to the frequency of occurrence of a microbe and the thickness of the edges corresponds to the frequency of occurrence of the two associated nodes. For example, Fig. 3a shows that the iterations of node 1 with nodes 2 and 3 respectively differ due to the width of the edges joining them (Dataset1). The thickness of the edges 1–2 is greater than that of 1–3 indicating that the number of times nodes 1 and 2 appeared together is higher than the number of nodes 1 and 3. The node size represents the frequency of node occurrence. This value reveals the association of microbes with specific diseases. Fig. 3a (Dataset 1) shows node 2 has the highest node size suggesting that there is more evidence in the literature supporting the association of the microbe with colonic cancer. Similar plots for Datasets 2 and 3 are provided in the Supplementary Figs. 6a and 7a.

A new network structure is then derived by infusing the clusters (Module 2) with the TSEA-computed network. The scores for each node in the network are then calculated by using the feature scores (Module 3). Fig. 3b shows the derived network's structure. The green edges indicate the associations between nodes which were found in the network (Fig. 3a). The red edges represent associations due to the clusters identified for Dataset 2 (Supplementary Figure 6b). The nodes 1,2 and 3 (Fig. 3b) form a single cluster and hence the edges 1–2, 2–3 and 3-1 are highlighted in red. The microbe with the highest node score doesn't necessarily have the highest node size. The node size captures the association of microbes with a particular disease; it does not, however, correspond to a direct correlation between them. The node score, on the other hand, corresponds to the association of the microbe with the output label. The two scores together aid the selection of microbes for targeted analysis. Similar plots for Datasets 2 and 3 are provided in the Supplementary Figs. 6b and 7b. It is easier to choose microbes in the case of Dataset 1 since the variance (σ2 = 2.35) of the node score allows for the identification of one helping microbe (*Prevotellaceae*). We note that this is not always the case. For example, the node scores for the Dataset 2 (Supplementary Figure 6b) exhibit limited variation (σ2 = 0.22) when compared to the Dataset 1 ones. For such cases, considering the node size along with node score, can potentially help the microbiome selection.

### 2.5 Performance comparison

#### 2.5.1 NFnetFU's performance comparison across different datasets

For both Datasets 1 and 2, we obtained a multi class AUC value of 0.5 and an AUC value of 0.477 was achieved for Dataset 3. Across the Datasets 1 and 2, the model predicts a constant value or response. We then performed binary class AUC for Dataset 3 but none of the combinations resulted in an AUC value more than 0.5.

#### 2.5.2 Microbe selection

**Fig. 4:**
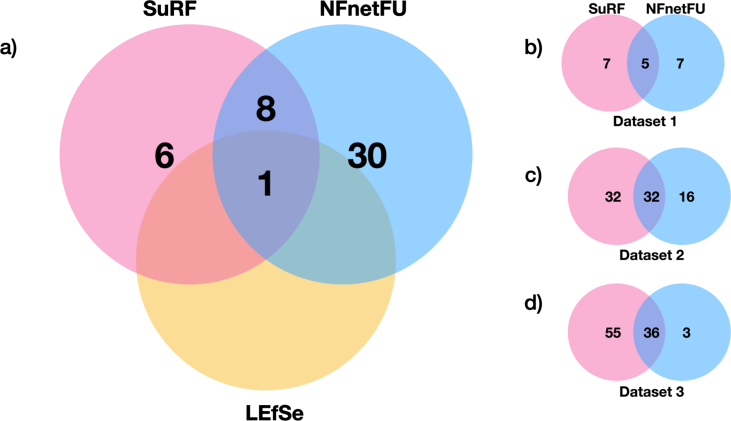
A Venn diagram elucidating the NFnetFU's (blue) performance analysis when compared to SuRF (pink) and LEfSe (yellow). a) A venn diagram showing overlapping microbes for all three methods for Dataset 3. Out of 39 microbes selected by NFnetFU, 15 were selected by SuRF and 1 was selected by LEfSe. Only one microbe was common amongst all methods. NFnetFU and SuRF adopt a similar approach for microbe selection which results in a larger overlap (9 microbes) between the two methods. SuRF also produces a rank list while performing the LASSO subsampling. The ranked OTUs were compared with the results from NFnetFU to gauge the overlap between the two methods for each dataset. b) SuRF and NFnetFU share 5 OTU and each also have 7 unique ones. c) SuRF and NFnetFU share 32 OTUs but SuRF ranks 32 additional OTUs whereas NFnetFU ranks 16 more apart from the 32 shared OTUs. d) SuRF and NFnetFU share 36 OTUs.

For the Datasets 1 and 2, no differentially abundant features were found in the first step at alpha 0.05 for the factorial Krushkal Wallis test among classes and threshold of 2.0 for the logarithmic LDA score. For the Dataset 3 however, the only potential biomarker selected wasLactobacillus. Similar to LEfSe no variables were selected using SuRF for Dataset 1 and 2. For the Dataset 3 however, SuRF selected 15 microbes with p values less than 0.05. Among the ones selected by SuRF, Lactobacillus, Bifidobacterium and Alistipes were the most significant microbes (p values close to zero), all 3 of which have appeared in NFnetFU priority list with Lactobacillus being prioritised above all the other microbes in the dataset by NFnetFU. Apart from the 3 microbes mentioned, 6 other microbes found in the results by SuRF were also found in NFnetFU results. Fig. 4a shows the overlap between the three methods.

#### 2.5.3 NFnetFU and SuRF OTU ranking

SuRF generated a list of ordered predictors that the LASSO variable selection has picked up with high frequency over observations subsamples. This list was then compared with the ranked list of most frequent microbes selected by NFnetFU. For Dataset 1 (Figure 4b), 5 out of the 12 OTUs, selected by both SuRF and NFnetFU, were common. *Distasonis*, which was prioritised by NFnetFU was also among the top 5 most frequent microbes depicted by the SuRF algorithm. For Dataset 2 (Fig. 4c), out of the 48 OTUs selected by the NFnetFu algorithm, 32 were also found in the SuRF selection. *Peptostreptococcus* was found, by SuRF, to be most frequently occurring amongst the other microbes and it was also assigned the highest priority by NFnetFU. For Dataset 3 (Figs. 4d), 36 of the 39 microbes selected by NFnetFU, were also selected by SuRF. Among these 36 microbes, Lactobacillus was dominantly prioritised by NFnetFU with the highest node score of 2.9 among the list of microbes. It was also selected as a viable biomarker by LEfSe.

## 3 Discussion

Microbiome data is inherently compositional, a characteristic that needs to be accounted for during any analysis so as to avoid misleading interpretations [37]. Sparse features can result in issues, such as overfitting, which affect the results of learning models. Such features, typically, increase the space and time complexity of the model, resulting in more fitted coefficients for regression models. Small effect sizes result in low signals which are difficult to detect. One of the major goal of regression analysis is to consolidate the relationship between dependent and independent variables. The regression coefficient, calculated as a resultant, represents the mean change in the dependent variable with respect to the independent variable. Collinearity weakens the statistical power of regression models by reducing the coefficient's precision. These problems render microbiome data analysis arduous. NFnetFU is a modularised, integrative pipeline that caters both microbiome data analysis, as well as a disease-specific microbe feature prioritization, that can potentially form the basis for hypothesis free research. It utilizes a number of modules that address inherent microbiome data analysis challenges. One of the novelties of NFnetFU lies with its adaptation of a neurofuzzy approach to convert the abundance dataset into a rule based matrix, emulating the behaviour of the original dataset with high accuracy. Commonly used analysis methods, such as the PhILR(Phylogenetic ILR) transform [47], allow for off-the-shelf statistical tools to be safely applied to microbiota surveys. However, data transformation as well as working within a ratio space are impeded due to the prevalence of zeros across 16S data (sparsity problem/excessive zero problem). We implemented a neuro-fuzzy inference system and employed a density-based clustering method to address the problems surrounding sparsity, collinearity, and effect size. Rigorous approaches, for example SparCC (Sparse Correlations for Compositional data) [48], applied to analyse correlation in microbiome datasets, also adopt a sparse data matrix. SparCC identifies correlations between taxa within ecological communities but the estimated correlation measures the linear relationship between log-transformed abundance. NFnetFU, on the other hand, captures non-linear patterns in microbiome datasets. Other methods, such as the LEfSe (Linear discriminant analysis Effect Size) [35], determine the features which are the most likely to explain the differences between classes. It combines standard tests for statistical significance with tests encoding biological consistency and effect relevance. Unlike NFnetFU, LEfSe adopts hard selection criteria for the features used in the analysis. It applies the Kruskall-Wallis test [49] so as to analyse all features and to assess whether the values are differentially distributed in different classes. Features violating the null hypothesis are subsequently analysed. The method is designed to identify differentially abundant features, as opposed to NFnetFU, which aims to prioritize microbes of interest. We use adaptive LASSO to approximate prioritization scores (referred to as “node scores”) for the microbes considered within our analysis. These scores capture the association of each microbe with the outcome variable. The algorithm also provides enrichment scores (referred to as “node sizes”, relating to the frequency of occurrence of a microbe (see method section)) indicating the association of the microbe with the specified disease. Together these scores provide an informative overview of the results and aid the selection of microbes for potential further targeted studies. They also provide a ranking for OTUs/microbes within a node, further enriching the results' interpretability. One of the biggest disadvantages of using NFnetFU lies with using ANFIS to produce a rule based matrix. As ANFIS primarily aims to reduce the error rate, it is very computationally expensive. Hence, it is necessary to employ a size reduction method and preprocess the data before using ANFIS. In this paper we use random forest-based feature selection method [50] but more sophisticated methods like Prototype Selection by Clustering (PSC) algorithm [51,52] would result in a more robust size reduction. Also, compared to methods such as the SPIEC-EASI (SParse Inverse Covariance Estimation for Ecological Association Inference) [53] which has been applied to predict previously unknown microbial associations, NFnetFU will only prioritize microbes from a given input list of known microbial interactions and hence cannot reveal previously unknown associations.

In this study, we used three datasets (Table 3) to analyse the performance of NFnetFU and we interrogated published literature for evidence to support our findings. For the first dataset we explored (Dataset 1), following the application of NFnetFU, we selected, based on the highest node score, *Prevotellaceae*. We found various reports in the literature indicating enrichment of the microbial community of the group, *Prevotellaceae*. Sun et al., 2016 [54] revealed a family enrichment of *Porphyromonadaceae* and *Prevotellaceae* within the inflammatory group, with a significant decrease in the hyperproliferation and adenoma groups (p < 0.01). Yang et al., 2019 [55], in an effort to elucidate the connection between gut microbiota, diet, and CRC, carriage of *Pseudomonadaceae*, *Moraxellaceae*, *Prevotellaceae*, and *Pasteurellaceae* reported significantly lower concentrations in the colorectal cancer patient group than that in the healthy control group at a family level (p < 0.05). For the second dataset we assessed (Dataset 2), we found *Erysipelotrichaceae* and *Clostridium* to be very closely ranked, based on their node score. Chen et al., 2012 [56] investigated intestinal microbiota, to capture the involvement of gut microbiota in the progression of colorectal cancer, and reported increased abundance levels of *Erysipelotrichaceae* in the lumen of colorectal cancer patients as compared to healthy controls. Kaakoush, 2015 [57] also documented the important role of *Erysipelotrichaceae* in human host physiology and/or disease. Roberts et al., 2014 [58] and Theys and Lambin, 2015 [59] discussed the ability of species of *Clostridium* bacteria to lyse tumor cells growing in hypoxic environments. Similarly, for the third dataset we employed (Dataset 3), *Lactobacillus* was ranked as the feature with the highest node score among the given microbes. *Lactobacillus* is a gut-resident probiotic beneficial to the host's health [60]. Zhuo et al., 2019 [60] discussed the association of *Lactobacillus acidophilus,* a member of the *Lactobacillus genus* of bacteria, to the development of colorectal cancer, and it's involvement in enhancing anti-tumor immunity in a mouse colon cancer model[61]

**Table 3:** Published datasets used for the NFnetFU analysis. The datasets are all related to colonic cancer studies but vary in size, feature type, and species.Table 3a discusses the studies and outcomes related to the different datasets and Table 3b discusses different dataset attributes, such as no. of samples, no. of features, feature type, etc.

3a)				
Dataset	Published dataset used	Outcome Comparison	Sample	Pubmed ID
1	Quraishi et al., 2020 [67]	Colonic biopsies were collected from patients with PSC-IBD, UC, and healthy controls	PSC-IBD (N = 10), UC (N = 10) and healthy controls (HC; N = 10)	PMID: 32016358
2	Sze et al., 2017 [68]	Before and after treatment for adenoma, advanced adenoma, and carcinoma	Adenoma (*N* = 22), Advanced adenoma (*N* = 19), and Carcinoma (*N* = 26).	PMID: 29145893
3	Zackular et al., 2015 [69]	Adult (8–12 week old) male mice were treated with all possible combinations of metronidazole (0.75 g/L), streptomycin (2 g/L), and vancomycin (0.5 g/L) to create eight treatment groups	no antibiotics (N = 12), all antibiotics (n = 9), metronidazole (n = 5), streptomycin (n = 5), vancomycin (n = 5), metronidazole only (N = 5), streptomycin only (N = 5), and vancomycin only (N = 3).	PMID: 27303681

3b)						
Dataset	No. of samples	No. of features	Feature Type	Species	Data Type	No. of outcome categories
1	30	47	OTU	Human	Processed	3
2	490	6393	Microbes taxa	Human	Raw Count	3
3	422	2606	OTU	Mice	Raw Count	8

LEfSe has a hard selection criteria in the first step (Krushkall - Wallis Test) which removes many OTUs considered as noise [35]. Although, LEfSe takes into account a dataset's sparsity and effect size, it overlooks high correlations across it. The relative difference, among classes depicted from the linear discriminant analysis model in LEfSe, is used to rank the features. Finally, a list of features discriminative with respect to the classes is generated. These features are further ranked according to the effect size with which they differentiate classes. The aim of the method is different when compared to that of NFnetFU. LEfSe focuses on ranking based on how efficiently features discriminate with respect to the classes whereas NFnetFu aims to rank features with respect to their association with the outcome variable. SuRF [62] is more advantageous in comparison to the existing methods for variable selection in terms of dealing with model inference and sparsity of selected models. Since its variable selection is based on a LASSO based approach, similar to the approach NFnetFU adopts, the results across the two tools are more comparable. NFnetFU was able to capture activity of many microbes shortlisted as important by SuRF. It also enriched the results with literally evidence supporting the involvement of microbes with the disease. Even though the AUC values indicate that the model is not predictive, addition of enrichment analysis makes the results biologically relevant.

Unlike previously discussed methods, NFnetFU takes into account a weighted cluster infused enriched network architecture to facilitate data interpretability, which in turn allows for an appropriate microbe selection, depending on the scope of potential microbiome targeted therapeutics studies. To demonstrate the NFnetFU's utility, we analysed the association of gut microbiota with inflammatory bowel diseases and colorectal cancer. Gut microbiome has recently been used as a biomarker for disease prognosis, phenotype based stratification, and response to treatment [62]. For instance, in the case of inflammatory bowel diseases, microbiome analysis has revealed important biomarkers for response to treatment and disease dysbiosis [63]. Other examples have revealed microbial metabolites (or enzymes) that play a role in disease progression, including pre-diabetes and type 2 diabetes [64], breast cancer [65] pancreatic cancer, etc. It remains fairly unclear how exactly the microbial community interacts with the host and how it participated in particular phenotype manifestations in diseases, such as cancer [66]. The application of NFnetFU allowed for the identification of specific microbiota catering for the opportunity of targeting and validating them in larger cohorts which forms a promising step for personalized medicine approaches.

In the future, we would like to explore the application of NFnetFU in longitudinal microbiome studies in an effort to identify microbes responsible for different time points. We would also like to enrich Module 4 by introducing directed networks or causal graphs. This will allow us to identify causal microbes and hence aid their prioritization for potential future translational microbiome research.

## 4 Conclusions

We developed a microbiome analysis framework that takes into account sparsity, collinearly and microbiome based enrichment analysis.

## 5 Materials and methods

### 5.1 Data description

We used three experimental datasets in our analysis. Dataset 1, published by Quraishi et al., 2020 [67], contains data related to colonic biopsies collected from patients with PSC-IBD (n = 10), UC (n = 10), and healthy controls (n = 10). In this study, the phenotypic differences between PSC-IBD and UC were assessed by applying an integrative approach over gut microbiota, immune infiltration and colonic gene expression data. Dataset 2, published by Sze et al., 2017 [68], is comprised of a collection of microbiota data related to a study comparing a 67 patient cohort diagnosed with carcinoma, adenoma and advanced adenoma before treatment. The study tested the alteration in the bacterial populations associated with normal and disease colon due to the treatment for adenoma or carcinoma. Finally, Dataset 3, published by Zackular et al., 2015 [69], involves data derived from studies that were conducted using adult male mice to observe perturbation in the microbiota with different combinations of antibiotics. Mice were treated with all of the possible combinations of metronidazole (0.75 g/liter), streptomycin (2 g/liter), and vancomycin (0.5 g/liter) to create the following eight treatment groups: no antibiotics (*n =* 12), all of the antibiotics (metronidazole, streptomycin, and vancomycin; *n* = 9), Δmetronidazole (streptomycin and vancomycin; *n* = 5), Δstreptomycin (metronidazole and vancomycin; *n* = 5), Δvancomycin (metronidazole and streptomycin; *n* = 5), metronidazole only (*n =* 5), streptomycin only (*n =* 5), and vancomycin only (*n =* 3). This study explored the role of the gut microbiota in colon tumorigenesis by using an inflammation-based murine model. After performing a 16S rRNA analysis for microbial profiling in each case, we employed our framework to process the microbiome abundance data. A summary of the published experimentally derived datasets used in this study is presented in Table 3. These datasets not only differ in size but also in terms of the feature types they contain as well as the species they refer to. For example, Dataset 1 contains mixed level microbial taxa as feature names as opposed to the operational taxonomic unit (OTU) used in Datasets 2 and 3. The Dataset 1 features are of the form x__ABC, where x represents the taxonomic rank and ABC represents the name of the classification. For example, *c__Gammaproteobacteria* corresponds to a class of bacteria *Gammaproteobacteria.* Contrary to this, Datasets 2 and 3 consist of features of the form OTUxxx, which correspond to a particular taxonomic unit. For example, *Otu000001* (Dataset 2) corresponds to the taxonomic characterization: *domain:Bacteria(100), phylum:Firmicutes(100), class: Clostridia(100), order:Clostridiales(100), family:Lachnospiraceae(100)* and *genus:Blautia(100)*. The numbers in brackets indicate the number of individual organisms in a particular category. Also, our framework is compatible with both preprocessed and raw count data. For example, Dataset 1 contains preprocessed data with positive float entries whereas Datasets 2 and 3 encompass microbiome abundance data with positive integer entries.

### 5.2 Data pre-treatment

The various datasets that were analysed differ in size (Table 3). To reduce the computational time required for their analysis, we derived pre-filtered features from Datasets 2 (containing 6393 features) and 3 (containing 2606 features) by applying a random forest-based feature selection method (51). Subsequently, the top 100 features, from a list of decreasing feature importance in each case, were selected so as to reduce the overall time complexity. The random forest-based feature selection was processed using the caret (v6.0.76) R package and the results are available at https://github.com/VartikaBisht6197/NFnetFu.

## 6 Methods

### 6.1 Module 1: ANFIS (adaptive neuro-fuzzy inference system)

In order to address the sparsity and different effect sizes in microbiome datasets, a supervised learning method employing the Adaptive Neuro-Fuzzy Inference System (ANFIS) [42], which is an artificial neural network [70], based on Takagi–Sugeno fuzzy inference system [71], is applied. The Takagi–Sugeno fuzzy inference system is based on a five-layered network architecture and benefits from an inference system corresponding to a set of fuzzy IF-THEN rules that approximate nonlinear functions. The algorithm outputs produce a rule-based integer matrix retaining intra-feature correlations [72]. The ANFIS algorithm processes the data using the frbs (v3.2-0) R package [73], which implements various learning algorithms based on fuzzy rule-based systems. The package is applied to learn a model using input data with labels (for example, the PSC-IBD, UC, and healthy controls samples available in the Dataset 1) using fuzzy rule-based systems. NFnetFU uses the default parameter settings for the near fuzzy learning algorithm. The algorithm uses a gaussian membership function which uses two parameters, namely the mean and variance parameters. It uses the least square method to perform the parameter learning. The ANFIS learning input matrix is an augmented matrix computed by concatenating the microbiome abundance matrix and numeric labels. The string labels for each dataset are first converted into numeric factors and then appended to the abundance matrix. After ANFIS learning, the resulting matrix corresponds to the numerical counterpart of the linguistic inference rules. Each row represents an inference rule involving all features as well as the outcome variable. Each of these rules are termed as *effective samples* and the resulting matrix is called an *inferred matrix*. The last column of the inferred matrix, termed *effective label*, indicates the inferred numeric values for each of the outcome variables. The features in the resultant matrix are called *effective features*. The columns of the inferred matrix are then centered and scaled (also called auto scaled). The output of the module is a set of effective labels and updated inferred matrices with effective samples in rows and effective features in columns.

### 6.2 Module 2: DBSCAN (Density-Based Spatial Clustering of Applications with noise) based clustering on microbiome data

DBSCAN (Density-Based Spatial Clustering of Applications with Noise) [43], a density-based nonparametric data-clustering algorithm, is then applied to cluster highly collinear features together. The algorithm processes the data using the *dbscan* (v1.1-5) R package [74], which is a faster reimplementation of several other DBSCAN density-based algorithmsdescribed by Ester et al. (1996) [43]. A user-specified epsilon (eps) neighbourhood is then generated and a user-specified minimum number of points (minpts) in a neighbourhood threshold is applied so as to identify the core, border and noise points estimating the density around each data points. The core points are then joined into clusters and each of the clusters is assigned to border points.

The algorithm requires two parameters, namely the epsilon (eps) and the minpts parameters. Epsilon is used to define a neighbourhood while the minpts parameter forms the minimum number of features required in a epsilon defined neighbourhood to form a cluster. We specify a minimum of two features required in a neighbourhood for the DBSCAN algorithm. To calculate the appropriate eps value, a list of possible eps values ranging from 1 to the maximum entry value of the inferred matrix, with a step size of 0.5, is passed as a parameter to a grid search. For a selected eps value, the algorithm computes the clustered matrix and fits a logistic regression to estimate the regression coefficients. It then checks for null values among the computed coefficients, which are indicative of a strong association between the matrix's features. When no further NAs are identified, for a given eps value, the process is terminated. Once the DBSCAN parameters are set, multiple sets of features, grouped together, are generated, termed as *clusters*. The features of the matrix can either correspond to a combination of features or a single feature. For each cluster, the module replaces the features of the input matrix's cluster with a new feature, which is a linear combination of the cluster features. The first PCA [75] loading, calculated for each feature in the cluster, is used as coefficient in the linear combination. The resulting matrix is termed a clustered matrix.

### 6.3 Module 3: adaptive LASSO (Least Absolute Shrinkage and Selection Operator) cluster scores

Scores are then calculated for all the features using the clustered matrix output. We use adaptive LASSO so as to calculate scores for all the clustered matrix's features, termed cluster scores. These cluster scores are then used to calculate the feature scores corresponding to individual features. Adaptive LASSO (Least Absolute Shrinkage and Selection Operator) [44] is a variation of the original LASSO technique [76] with oracle properties i.e. simultaneous, consistent variable selection [45] and optimal variable estimation. This is achieved by assigning data-driven weights to different coefficients, while penalizing them by a ℓ₁ penalty, according to the original LASSO method. These weights represent the absolute value of the coefficients derived by fitting a generalized linear model to scaled input data. The data is subsequently scaled again, with respect to these calculated weights, so as to calculate initial betas. The initial betas are calculated via a k-fold cross-validation using glmnet. Glmnet (v4.0-2) [77] is an R package that fits the entire lasso or elastic-net regularisation path for various regression models. The following formula is applied to calculate adaptive LASSO coefficients. Here, w corresponds to weights and β corresponds to the initial betas associated with the respective features.

$${\mathrm{argmin}}_{\beta }∥y-\sum_{j=1}^{p}x_{j}\beta_{j}{∥}^{2}+\lambda \sum_{j=1}^{p}w_{j}|\beta_{j}|$$

The coefficients or cluster scores are associated with either individual features or a combination of features. To calculate the feature score, based on a given cluster score for a particular combination of features, we use the loading values derived by Module 2.

### 6.4 Module 4: network fusion using enrichment analysis and visualization

#### 6.4.1 Cluster-infused TSEA based network architecture

The Taxon Set Enrichment Analysis (TSEA) module of MicrobiomeAnalyst [32,33] is a web-based platform that analyse common data outputs from current microbiome studies comprehensively. Further it is used to test whether there are enrichments of taxon sets for a list of microbes of interest. MicrobiomeAnalyst, including its underlying R code, is freely accessible as a web-based application [78]]. MetaboAnalyst is part of a suite of metabolomics databases that includes the Human Metabolome Database (HMDB) [[79], [80], [81]] DrugBank [[82], [83], [84]], Toxin and Toxin-Target Database [85], as well as [86]. Given a list of microbes of interest, TSEA is applied to assess whether there are enriched mixed-level taxon sets that have been identified to be significantly associated with particular developmental, physiological, or disease conditions. Table 3 lists the datasets, and the different types of information associated with them, used. For example, the Dataset 1 features correspond to mixed level taxons whereas the Dataset 2 and 3 features are taxon specific (OTUs). These features are converted into the exact microbe names used in the TSEA database. This part of the analysis is not automated and depends on the input file, i.e., whether the feature names are mixed level taxa or OTUs with associated taxonomical files. Various microbes are joined together based on their converted microbe name. For example, the TSEA name *Myxococcales* (Dataset 1) is based on the combination of two features, namely *f__Myxococcales.0319.6G20* and *s__Myxococcales.sp*. These features are combined together in a cluster (Module 2), using an independent method which also validates the combination. The immediate parent level classification is used for the features of an unclassified taxonomic level. For example, *Otu00002* (Dataset 3), corresponding to the taxonomy domain*:Bacteria*(100), phylum*:Proteobacteria*(100), class*:Gammaproteobacteria*(100), *Enterobacteriales*(100), order:*Enterobacteriaceae*(100), genus*:unclassified*(100), was converted to *Enterobacteriaceae* since its genus level was unclassified. TSEA interrogates a database library and lists instances of associations for a given set of mixed taxon level microbes for particular diseases. The TSEA results include a reference to associated diseases, studies, as well as to other associated microbes, their taxonomic classification, etc. The results are then filtered based on a disease of interest.

The module considers two criteria, namely the frequency of occurrence of individual microbes and the frequency of a pair of microbes co-occurrence within a study, for the construction of a network. An adjacency matrix [87], accounting for these criteria, was then calculated and used to compute the network. The adjacency matrix is a square matrix with the rows and columns corresponding to the microbes. The diagonal entries of the adjacency matrices indicate the frequency of occurrence of individual microbes and the other entries indicate the frequency of occurrence of the associated pair of microbes in a study together. For computing the network, only the microbes which appeared at least once within the results subset were considered. The size of the nodes in the network corresponds to the occurrence of individual microbes, whereas the thickness of edges corresponds to the co-occurrence of the associated pair of microbes in a study. The microbes included in the network are termed *microbial nodes*. Features associated with microbial nodes are combined, based on their taxonomic similarities.

We then first derive a new network structure by incorporating identified clusters (Module 2) in the existing structure and then we use the feature scores (Module 3) to compute the node scores for each node in the network. All of the identified clusters (Module 2) are assessed in terms of their validity and then added to the network. A cluster is considered valid if any one of the features belonging to the cluster is associated with the microbial nodes. For instance, Fig. 3a represents a biological network (Dataset 1) with the microbial node 7 (*Lentisphaeria*) corresponding to *c__Lentisphaeria,* clustered with *p__Lentisphaerae* and *o__Victivallales* (identified by Module 2). Thus, the Module 2 derived cluster, namely the *c__Lentisphaeria, p__Lentisphaerae* and *o__Victivallales*, is considered as a valid cluster. Additional nodes, for all the valid considered clusters, *p__Lentisphaerae,* and *o__Victivallales* in this case, are then added to the existing network and the new nodes are assigned a node size of 1. All the edges joining the nodes of valid clusters are presented in red. Fig. 3b represents an example of such a cluster-infused TSEA based network architecture. Nodes 1, 2 and 3, corresponding to class:*Gammaproteobacteria,* family:*Enterobacteriaceae* and order:*Enterobacteriales,* form a cluster (Module 2). This is a valid cluster since one or more members of the cluster are present in the network. Hence, the edges joining the node are depicted in red. Other valid clusters identified within the same dataset, for example the *f__Myxococcales.0319.6G20* and *s__Myxococcales.sp* cluster, are not shown explicitly since their combination forms the microbial node *Myxococcales*. The g__Rothia and s__mucilaginosa cluster (Dataset 1) is an example of an invalid cluster as none of the members of the cluster are present in the network.

The node scores for each microbial node is calculated sum over xi and divided by “n” where xi represents the feature score for the ith feature in the set of *n* features associated with the microbial node.

For example, so as to calculate the node score for *Myxococcales* (Dataset 1), we use the feature scores of *f__Myxococcales.0319.6G20* and *s__Myxococcales.sp*. The node score and node size together help the microbe prioritization within the network.

#### 6.4.2 Comparison of NFnetFU with other methods

We compared the performance of NFnetFU with the performances of SuRF (Subsampling ranking forward selection) and LefSe (Linear discriminant analysis Effect Size). The comparison was made based on three criteria, namely performance measure using the area under curve (AUC), QUTs automatic and third, OTUs ranking.

#### 6.4.3 SuRF

This method includes subsampling and forward-selection methods which primarily focus on microbiome analysis. The SuRF [62] framework consists of mainly two steps. Firstly, an ordered list of predictors, using the LASSO variable selection method, is generated over subsampled observations. A forward selection, along with ANOVA, is then applied to the variable list. Finally, using likelihood ratios, the significance of each variable is calculated in the forward selection.

#### 6.4.4 LefSe (linear discriminant analysis effect size)

LEfSe (Linear discriminant analysis Effect Size) [35] is an algorithm for high-dimensional biomarker discovery primarily employed in microbiome research studies. LEfSe first identifies features that are statistically different among the outcome variable (for example: control vs. CRC patients). It then performs additional tests to assess whether these differences are consistent with respect to the expected biological behaviour. A Krushkal Wallis test is then performed followed by a wilcox test. Finally, a linear discriminate model is generated which ranks the features based on their relative differences between classes.

#### 6.4.5 R scripts and functions

We used the R (https://www.r-project.org) v4.0.0.0 software for statistical computing and all related scripts and all the algorithms that are part of our framework are freely available at. https://github.com/VartikaBisht6197/NFnetFu. An Rmarkdown manual describing inputs and outputs for each module can be found at https://rpubs.com/Vartika/760624 (Dataset 1).

## Contributions

VB performed the analysis and developed the framework. AA conceived and designed the microbiome data analytics strategy; AA and GVG supervised the study; all authors contributed to the results interpretation, co-wrote, edited and reviewed the manuscript. All authors read and approved the final manuscript.

## Funding acknowledgement

The authors acknowledge support from support from the NIHR Birmingham ECMC, NIHR Birmingham SRMRC, Nanocommons H2020-EU (731032) and the NIHR Birmingham Biomedical Research Centre and the MRC Health Data Research UK (HDRUK/CFC/01), an initiative funded by 10.13039/100014013UK Research and Innovation, Department of Health and Social Care (England) and the devolved administrations, and leading medical research charities. The views expressed in this publication are those of the authors and not necessarily those of the NHS, the National Institute for Health Research, the Medical Research Council or the Department of Health.

## Ethics approval and consent to participate

Not applicable.

## Consent for publication

Not applicable.

## Declaration of competing interest

The authors declare that they have no competing interests.

## Figures and Tables

**figs1:**