# Supplementary figures and images for: NFnetFu: A novel workflow for microbiome data fusion
Source: Comput Biol Med. 2021 Aug;135:104556. doi: 10.1016/j.compbiomed.2021.104556 (PMC8404037; doi:10.1016/j.compbiomed.2021.104556)

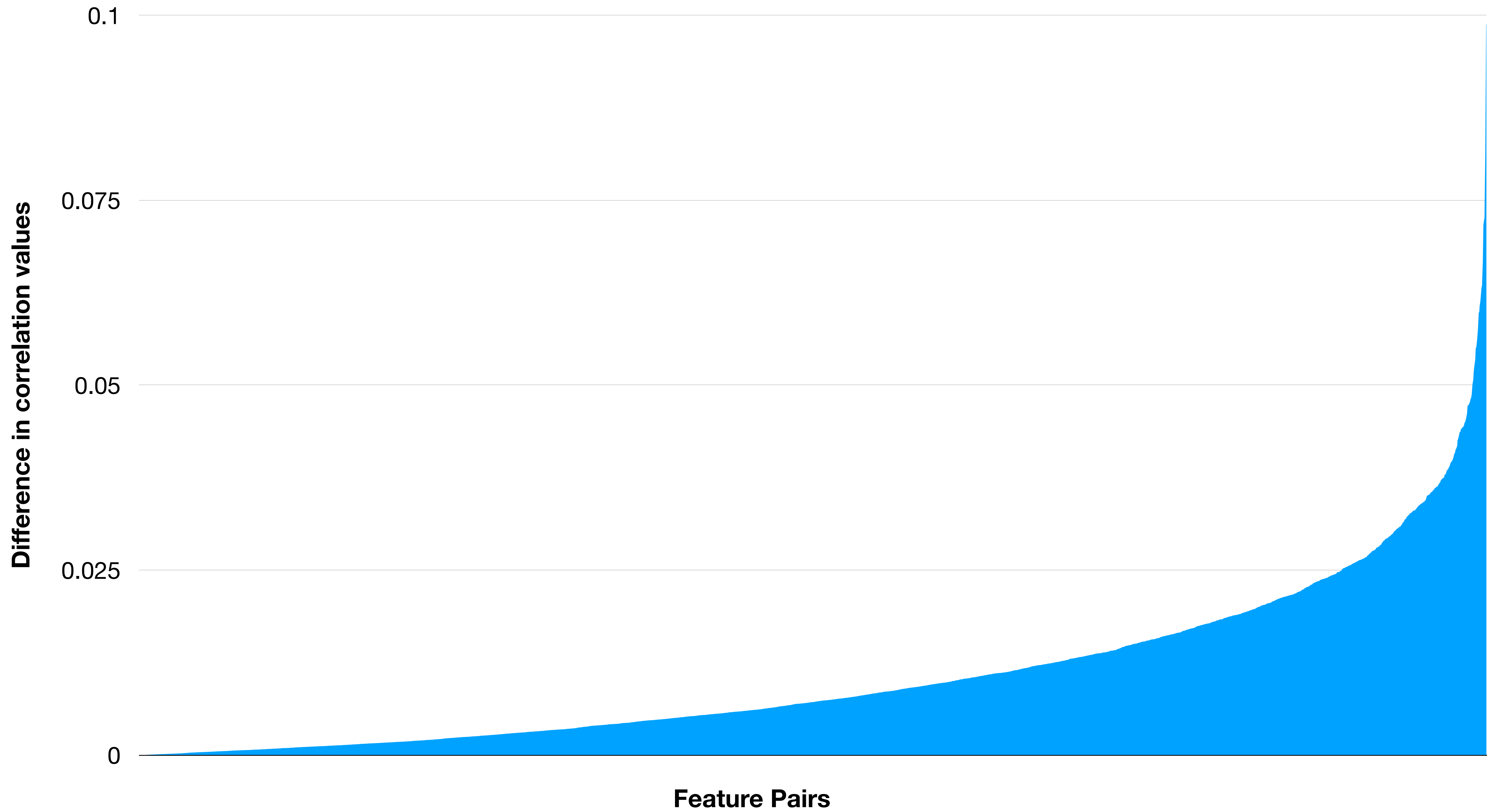

Supplement: Multimedia component 1 [file mmc1.pdf]

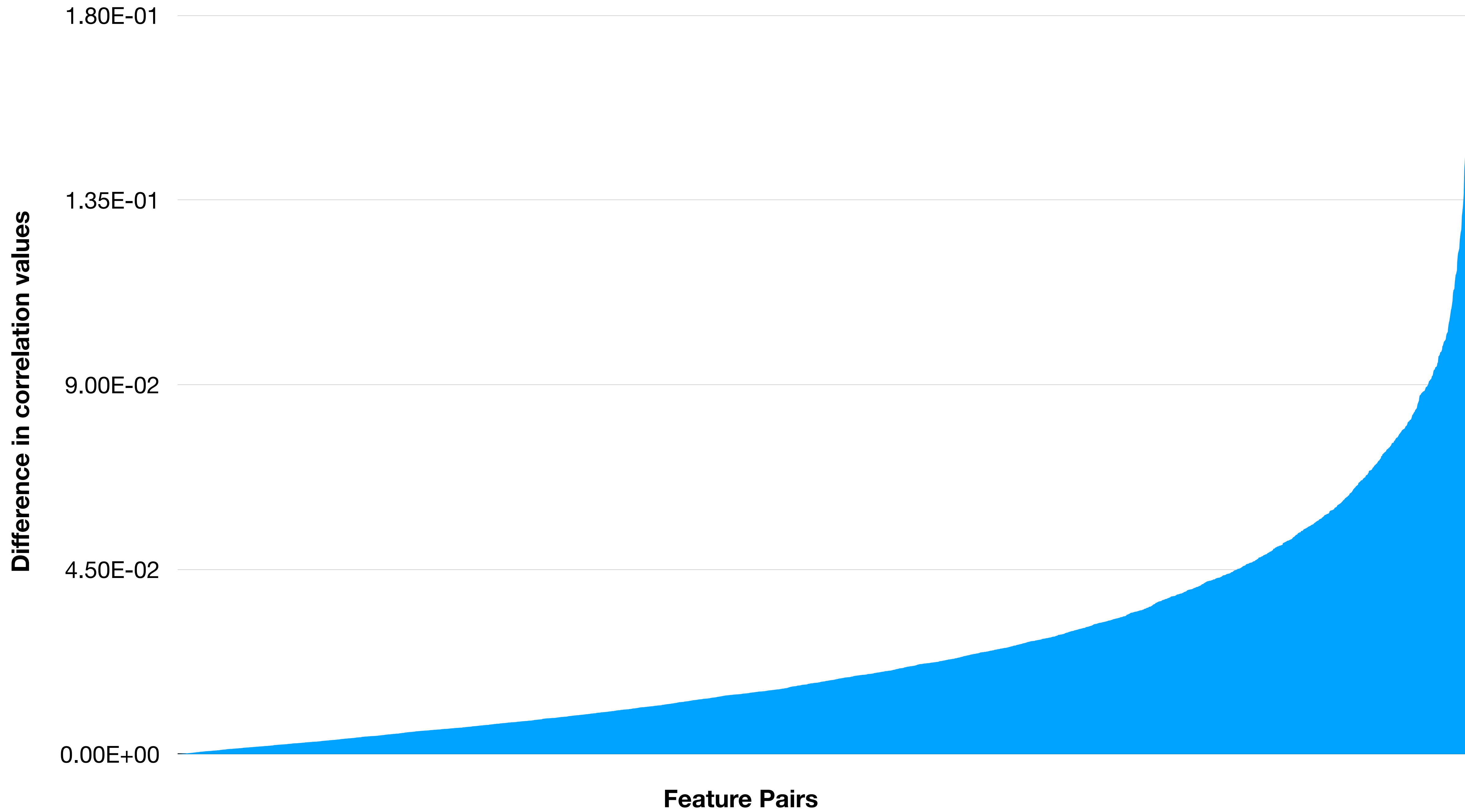

Supplement: Multimedia component 2 [file mmc2.pdf]

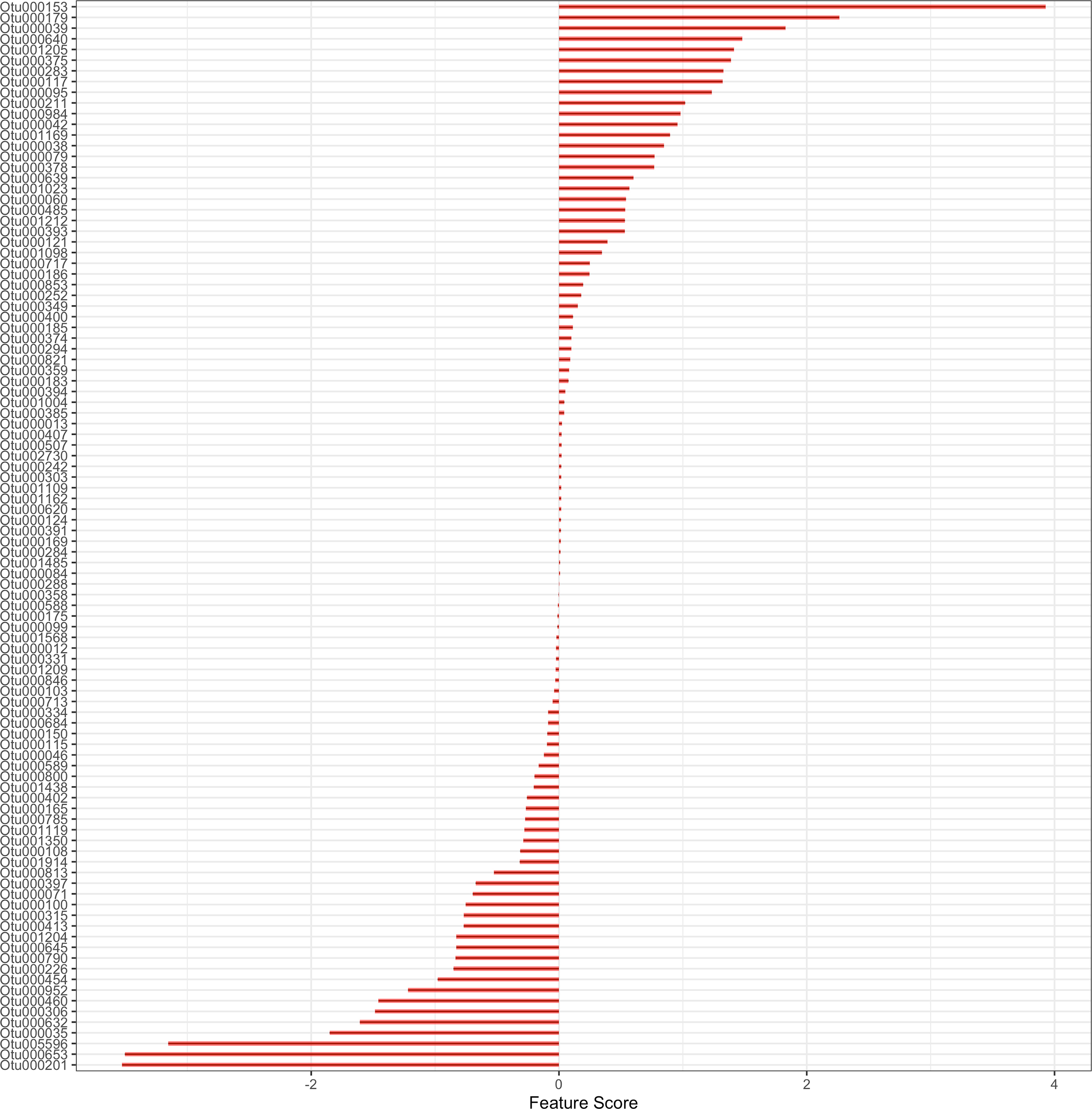

Supplement: Multimedia component 26 [file mmcfigs2.jpg]

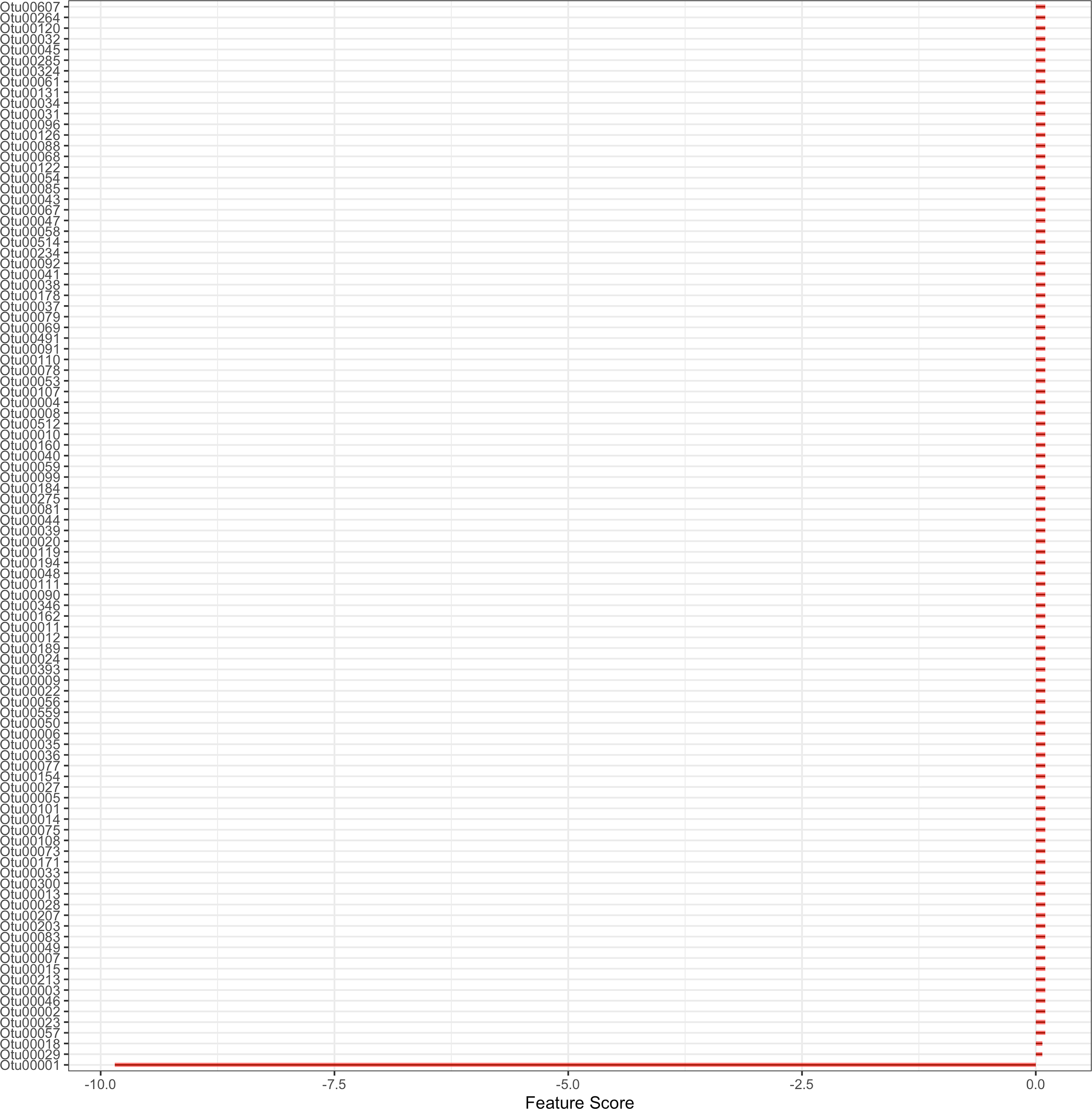

Supplement: Multimedia component 27 [file mmcfigs3.jpg]
