# Supplementary material for: NFnetFu: A novel workflow for microbiome data fusion
Source: Comput Biol Med. 2021 Aug;135:104556. doi: 10.1016/j.compbiomed.2021.104556 (PMC8404037; doi:10.1016/j.compbiomed.2021.104556)
Supplement: Multimedia component 17 [file mmc17.pdf]

a)

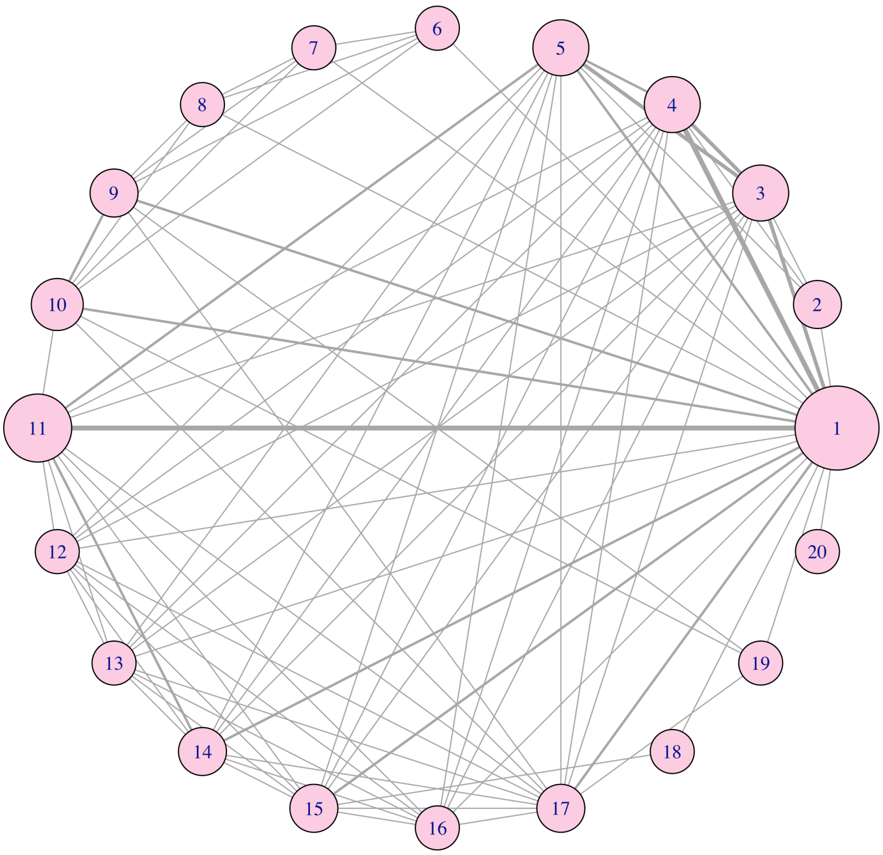

Node	Microbe Names	Node Size
**1**	Bacteroides	11
**2**	Lactobacillus	2
**3**	Bifidobacterium	4
**4**	Ruminococcus	4
**5**	Streptococcus	4
**6**	Enterobacteriaceae	1
**7**	Bacteroidales	1
**8**	Enterococcus	1
**9**	Firmicutes	2
**10**	Clostridiales	3
**11**	Prevotella	7
**12**	Burkholderiales	1
**13**	Parabacteroides	1
**14**	Alistipes	2
**15**	Odoribacter	2
**16**	Lachnospiraceae	1
**17**	Ruminococcaceae	2
**18**	Akkermansia	1
**19**	Bacteroidetes	1
**20**	Bacillales	1

b)

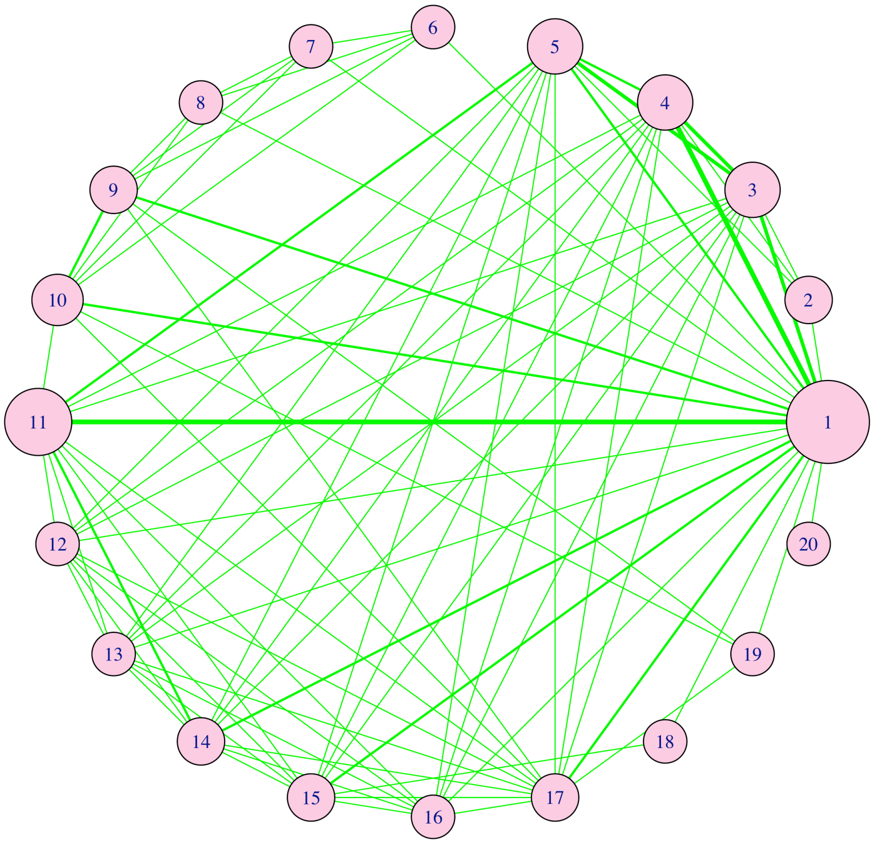

Node	Microbe Names	Node Size	Node Score
**1**	Bacteroides	11	0.1662
**2**	Lactobacillus	2	3.3075
**3**	Bifidobacterium	4	0.5452
**4**	Ruminococcus	4	0.1087
**5**	Streptococcus	4	0.0915
**6**	Enterobacteriaceae	1	0.0924
**7**	Bacteroidales	1	0.0930
**8**	Enterococcus	1	0.0929
**9**	Firmicutes	2	0.0651
**10**	Clostridiales	3	0.0867
**11**	Prevotella	7	0.1797
**12**	Burkholderiales	1	0.1625
**13**	Parabacteroides	1	0.1046
**14**	Alistipes	2	0.0940
**15**	Odoribacter	2	0.0948
**16**	Lachnospiraceae	1	0.0831
**17**	Ruminococcaceae	2	0.0925
**18**	Akkermansia	1	0.0486
**19**	Bacteroidetes	1	0.0950
**20**	Bacillales	1	0.1008
