# Supplementary material for: NFnetFu: A novel workflow for microbiome data fusion
Source: Comput Biol Med. 2021 Aug;135:104556. doi: 10.1016/j.compbiomed.2021.104556 (PMC8404037; doi:10.1016/j.compbiomed.2021.104556)
Supplement: Multimedia component 19 [file mmc19.pdf]

a)

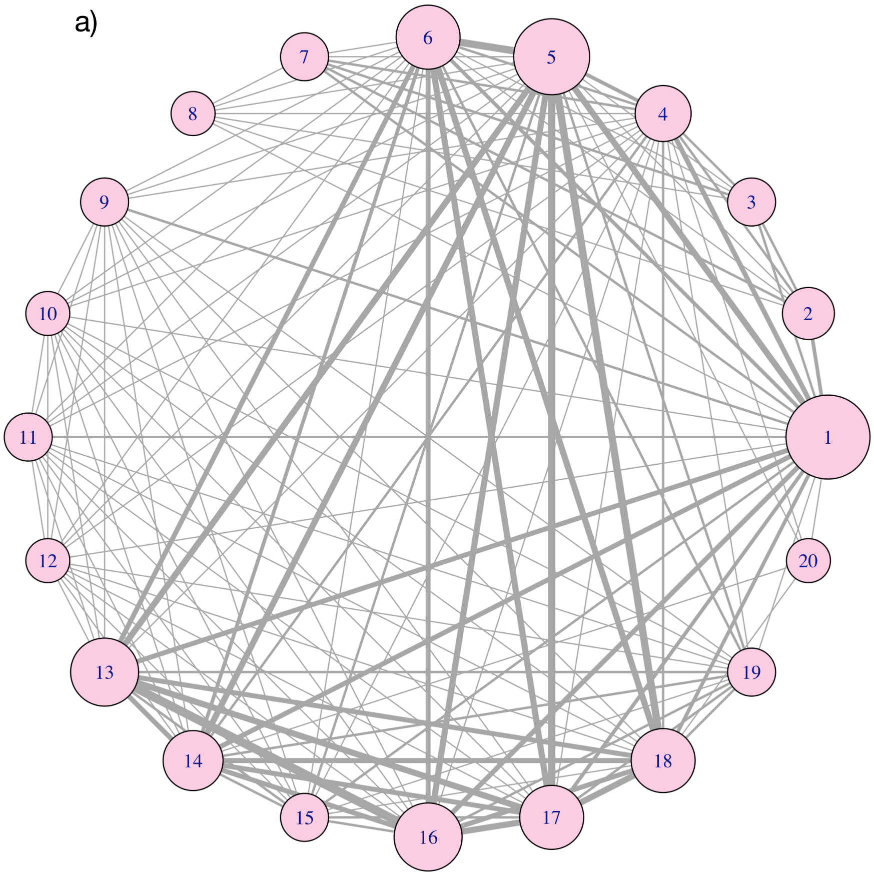

Node	Microbe Names	Node Size
**1**	Bacteroides	11
**2**	Blautia	3
**3**	Coprococcus	2
**4**	Ruminococcus	4
**5**	Fusobacterium	9
**6**	Gemella	6
**7**	Roseburi	2
**8**	Eikenell	1
**9**	Ruminococcaceae	2
**10**	Lachnospiraceae	1
**11**	Odoribacter	2
**12**	Bilophila	1
**13**	Prevotella	7
**14**	Porphyromonas	5
**15**	Alistipes	2
**16**	Prevotell	7
**17**	Parvimonas	6
**18**	Peptostreptococcus	6
**19**	Dialister	2
**20**	Dore	1

b)

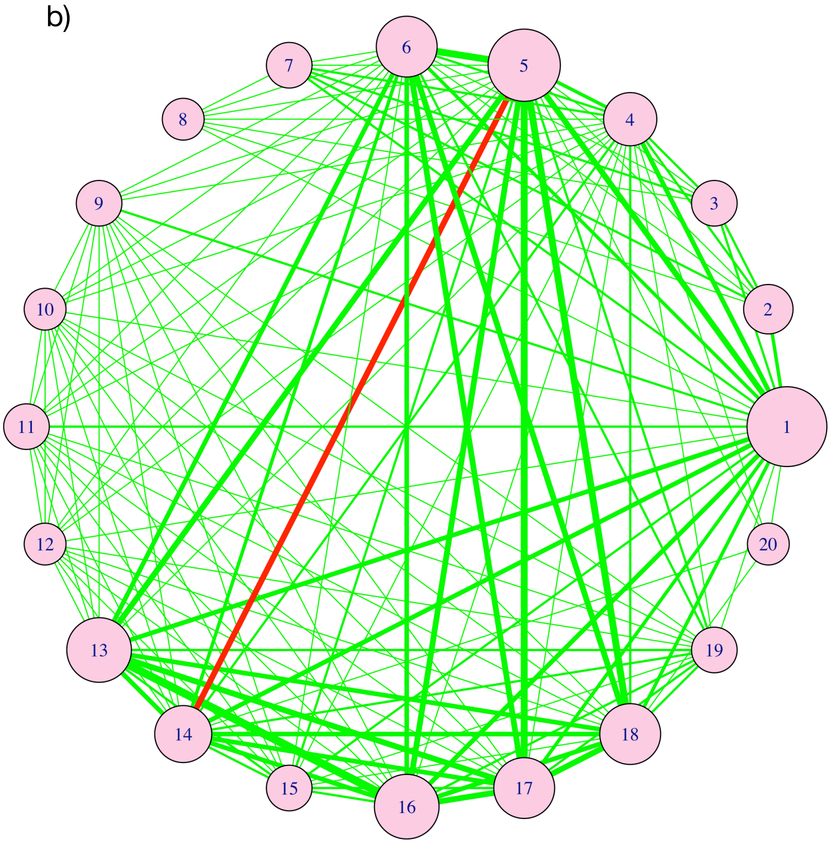

Node	Microbe Names	Node Size	Node Score
**1**	Bacteroides	11	0.4455
**2**	Blautia	3	0.0528
**3**	Coprococcus	2	1.0362
**4**	Ruminococcus	4	0.1100
**5**	Fusobacterium	9	0.8696
**6**	Gemella	6	0.7156
**7**	Roseburi	2	0.9110
**8**	Eikenell	1	0.4279
**9**	Ruminococcaceae	2	0.7437
**10**	Lachnospiraceae	1	0.7864
**11**	Odoribacter	2	1.0081
**12**	Bilophila	1	0.9810
**13**	Prevotella	7	0.8525
**14**	Porphyromonas	5	2.2906
**15**	Alistipes	2	0.0555
**16**	Prevotell	7	1.3241
**17**	Parvimonas	6	1.2530
**18**	Peptostreptococcus	6	2.5150
**19**	Dialister	2	0.6907
**20**	Dore	1	0.7271
